# Special Issue: Latest Advances in Delivery and Outcomes of Cardiac Resynchronization Therapy and Conduction System Pacing

**DOI:** 10.3390/jcm12103453

**Published:** 2023-05-14

**Authors:** Alexander H. Maass, Fenna Daniëls, Eva Roseboom, Kevin Vernooy, Michiel Rienstra

**Affiliations:** 1 Department of Cardiology, University Medical Center Groningen, University of Groningen, 9700 RB Groningen, The Netherlands; 2 Department of Cardiology, Isala Hospital, 8000 GK Zwolle, The Netherlands; 3 Department of Cardiology, Cardiovascular Research Institute Maastricht (CARIM), Maastricht University Medical Center, 6202 AZ Maastricht, The Netherlands

Cardiac Resynchronization Therapy (CRT) is an established technique to improve morbidity and mortality in selected heart failure patients. This technique has been a game changer for heart failure treatment in patients with conduction delay, who are often non-responders to optimal medical treatment. Unfortunately, rates of non-response to CRT remain at 30% to 50% depending on the outcome measure that is considered. Non-response rates are due to suboptimal delivery of CRT, patient selection, and device programming. Furthermore, there is still controversy on which measure and cut-off should be used to define the CRT response. There are abundant “gaps in the evidence” that need to be filled by prospective studies or analyses of retrospective cohorts. We describe some of the recent developments and coin some of the areas where we expect to receive new insights in the near future.

Since we published our previous special issue on CRT (https://www.mdpi.com/journal/jcm/special_issues/Cardiac_Resynchronization_Therapy, accessed on 29 April 2023), cardiac conduction system pacing (CSP) has evolved as an alternative to CRT. CSP is mainly used when the delivery of CRT is not achieved or in non-responders to CRT [1]. The transition of CSP to first-line therapy instead of CRT awaits landmark trials, despite a significant number of centers already using CSP as a replacement for CRT [2,3]. The early adoption of innovations is of importance for patients; however, clinical trials need to be performed at the same time to make sure that we learn about CSP in a structured way and learn more about its weaknesses. In addition to outcome studies, we still need to learn more about the optimal implantation technique. The development of specific tools, electrodes, and pacemaker programming will hopefully improve CSP delivery in the near future.

With this Special Issue, we would like to highlight recent advances to improve the outcomes of patients treated with CRT, including, but not restricted to, strategies to optimize implantation techniques, patient selection, or CRT/CSP programming. We encourage researchers to submit their findings on how to provide optimal CRT/CSP. The measurement of outcome variables such as exercise capacity, quality of life, reverse remodeling, as well as the occurrence of atrial and ventricular arrhythmias is essential to evaluate the success of this powerful treatment. We especially encourage investigators to provide their experiences with CSP in regard to these outcome parameters. In this Editorial, we would like to highlight some of the areas that we aim to target with this Special Issue.

The best way to define and evaluate the response to CRT has not yet been established. Whereas improving quality of life is the goal of most therapies, improving prognosis by reducing mortality and heart failure hospitalizations is the most utilized end point in most randomized heart failure therapy trials. A few studies have compared different definitions [4]. Reverse left ventricular remodeling is thought be the best predictor for these cardiovascular endpoints. It has been suggested, however, that left atrial reverse remodeling as a possible marker for atrioventricular resynchronization should be included in follow-up examinations [5,6,7]. The amount of reverse remodeling that defines a responder is also a subject of debate. Whereas a 10% reduction in left ventricular end-systolic volume might be the maximum in male patients with ischemic heart failure and renal disease, a 15% reduction might be a poor result in female patients with non-ischemic heart failure and typical left bundle branch block (LBBB). The amount of reverse remodeling can be predicted using a simple score including age, vectorcardiographic QRS area, and two simple echocardiographic parameters, interventricular mechanical delay, and apical rocking [8]. It has been suggested that age affects the outcome of CRT [9]. We have learned a lot about patient selection with a focus on ECG criteria. Echocardiography beyond the simple described parameters has been disappointing [10]. We might have to think outside the box to find new echocardiographic predictors such as right ventricular strain and pulmonary artery pressure [11]. Female patients seem to respond better to CRT, but it has been questioned whether this is merely due to differences in heart failure etiology and true left bundle branch block [12]. In addition to having a different response to CRT, inclusion criteria might have to be different in women. Furthermore, CRT programming should be tailored to the intrinsic atrioventricular delay, which is shorter in women [13]. Landmark trials included patients mainly in functional class II or III. Patients without any heart failure symptoms might also derive benefit from this [14]. It is questionable whether patients in functional class IV can respond to CRT or should receive work-up for mechanical support. If they respond to inotrope therapy, CRT could possibly delay or replace advanced heart failure therapies such as left ventricular assist devices or transplantation. Guidelines have changed significantly in the last 15 years [15]. Diminished renal function is a comorbidity that is associated with poor outcome in heart failure and might hamper the benefit of CRT [16]. On the other hand, it has been shown that an even lower amount of reverse remodeling is associated with a better outcome in CRT patients with renal insufficiency [17]. Non-ischemic dilated cardiomyopathy is associated with a higher degree of reverse remodeling. Patients with ischemic heart failure, however, show a beneficial response to CRT at a lower amount of reverse remodeling [18].

It is still a matter of debate whether a defibrillator should be added to CRT in most patients receiving this therapy. In particular, patients with non-ischemic cardiomyopathy and typical LBBB have a low residual arrhythmic risk after CRT. In addition to morphologic remodeling, CRT can induce electrical reverse remodeling [19]. In some patients, in particular in ischemic heart failure, CRT can be pro-arrhythmic [20]. The DANISH study and meta-analyses have demonstrated that there is additional value of adding defibrillator function in this group [21,22]. An additional debate is whether to downgrade super-responders to CRT at the time of battery depletion of their CRT-D device, as there remains a small but non-trivial arrhythmic risk [23,24].

Patients with permanent atrial fibrillation (AF) have not been included in most landmark clinical trials besides RAFT. In the latter study, patients with AF had no benefit from CRT [25]. After CRT, it is crucial to preserve a high percentage of biventricular pacing, as close to 100% as possible. In patients with AF, biventricular pacing percentages are underestimated by device diagnostics and atrioventricular junction ablation should be considered early in the course if rhythm control is not deemed feasible [26]. If rhythm control is feasible, pulmonary vein isolation has been shown to improve CRT response [27]. Patients with a high percentage of premature ventricular contractions should be considered for ablation [28].

An upgrade from right ventricular pacing is one of the major indications for CRT implantation even if evidence is scarce [29]. In a meta-analysis, the benefit of upgrades seems to be similar to de novo implantations [30]. In the only landmark study that allowed paced patients to be included, the RAFT trial, in the paced subgroup, no benefit of CRT was observed [31]. We eagerly await the results of the BUDAPEST-CRT study, which is the first randomized controlled study on CRT upgrades [32]. Upgrade procedures are generally technically more demanding and are associated with a high rate of complications [33].

Even after more than 20 years of CRT, lead delivery is still a matter of debate. What is the optimal lead position? The area of latest mechanical activation can be defined by echo or MRI, but this is not easily translated to implantation fluoroscopy [34,35]. The reduction in electrical dyssynchrony as assessed by reduction in vectorcardiographic QRS area could be an attractive target for lead placement strategies [36]. More simple to use would be QRS narrowing by CRT [37]; however, super-response can occur in the absence of a significant reduction in QRS duration. Reaching an optimal lead position can be difficult, but improved technologies such as snare techniques have increased the number of target veins within the coronary sinus [38].

Optimal CRT programming is also not yet perfectly defined. Whereas most implanters use a “one size fits all” programming algorithm, it is evident that this might work for most but certainly not all patients. It has been suggested that in order to reduce the number of non-responders but also to improve response in the whole group, an individualized approach is needed [39]. This strategy should probably involve not only cardiac device specialists but also heart failure cardiologists [40].

When it comes to conduction system pacing, most of the gaps in the evidence for CRT hold true, but additional areas of investigation need to be defined. Several small randomized controlled trials comparing CRT and CSP are recruiting patients and the first small studies have been published. Due to its limitations in terms of implantation success, durability, pacing threshold, and sensing signals, His bundle pacing has lost most of its ground when compared to left bundle branch are pacing (LBBAP) [41]. The best technique for LBBAP, however, has not been defined. It has been shown in a large registry with more than 2500 patients that the predominant LBBAP capture type was left bundle fascicular capture, followed by left ventricular septal capture, and few patients had proximal left bundle branch capture [42]. In addition, in this study, the implantation success was only 82% in heart failure patients. Whether CSP is a valid option for all patients that are currently implanted with CRT is yet to be addressed. Patients that are indicated for upgrading from RV pacing are an attractive target even though, as mentioned before, CRT efficacy has not been thoroughly defined in this patient group [43]. Optimal programming for CSP has not been defined. The best spot for CSP is also a matter of debate (His bundle vs. left bundle branch vs. fascicular pacing) as well as the long-term effect of right bundle branch block on right ventricular function.

In summary, there are many gaps in the evidence for CRT and even more for CSP. We hope this Special Issue will contribute to the knowledge about CRT and CSP, although we realize that most studies create new questions even when they do provide some answers.
